# Development of core outcome sets of Food for Special Medical Purposes designed for type 2 diabetes mellitus: a study protocol

**DOI:** 10.1186/s13063-023-07214-2

**Published:** 2023-03-24

**Authors:** Dongyu Mu, Jie Gong, Yaoyao Wei, Muxi Chen, Jiajie Yu, Liang Du, Wen Hu

**Affiliations:** 1 Department of Clinical Nutrition, West China Hospital, Sichuan University, #37 Guoxue Alley, Wuhou District, Chengdu, 610041 Sichuan Province People’s Republic of China; 2 West China School of Public Health, Sichuan University, Chengdu, 610041 People’s Republic of China; 3 West China School of Medicine, Sichuan University, Chengdu, 610041 People’s Republic of China; 4 The Chinese Evidence-Based Medicine Center, West China Hospital, Sichuan University, Chengdu, 610041 People’s Republic of China; 5 West China Medical Publishers, West China Hospital, Sichuan University, Chengdu, 610041 People’s Republic of China

**Keywords:** Food for special medical purposes, Clinical trials, Core outcome sets, Type 2 diabetes mellitus

## Abstract

**Background:**

The Chinese government stipulates all food for special medical purposes (FSMP) designed for specific diseases to be tested in clinical trials before approving it for registration. The process of developing core outcome sets (COSs), the minimum sets of outcomes supposed to be measured and reported, provides an economical and practical option for stakeholders to communicate and cooperate in conducting clinical trials as well as in reporting FSMP outcomes. This study uses type 2 diabetes mellitus (T2DM) as an example to develop COS for clinical trials of FSMP.

**Methods:**

The COS for FSMP-T2DM will be divided into 3 phases and developed following COS-STAP and COS-STAD:

(1) Generate a list of relevant outcomes identified from a systematic review, in which information sources will mainly include published studies, regulatory documentation, and qualitative interviews of stakeholders. The identified outcomes will be categorized using a conceptual framework and formatted into the first round of the Delphi survey questionnaire items.

(2) At least 2 rounds of Delphi surveys will be performed among stakeholders to create the COS for FSMP-T2DM. Patients, clinical dietitians, physicians, COS researchers, journal editors, FSMP manufacturers, and regulatory representatives will be invited to score each outcome from aspects of importance.

(3) Hold a face-to-face or online consensus meeting to refine the content of the COS for FSMP-T2DM. Key stakeholders will be invited to attend the meeting to discuss and agree on the final COS.

**Discussion:**

We have prepared an alternative solution of the Likert scale selection, Delphi survey rounds, scoring group, and consensus definitions in case of an unexpected situation.

**Trial registration:**

COMET (1547). Registered on March 23, 2020.

**Supplementary Information:**

The online version contains supplementary material available at 10.1186/s13063-023-07214-2.

## Background

This study protocol follows the Core Outcome Set-STAndardised Protocol Items (COS-STAP), a checklist of 13 items considered essential documentation in a COS protocol [1].

**Fig. 1:**
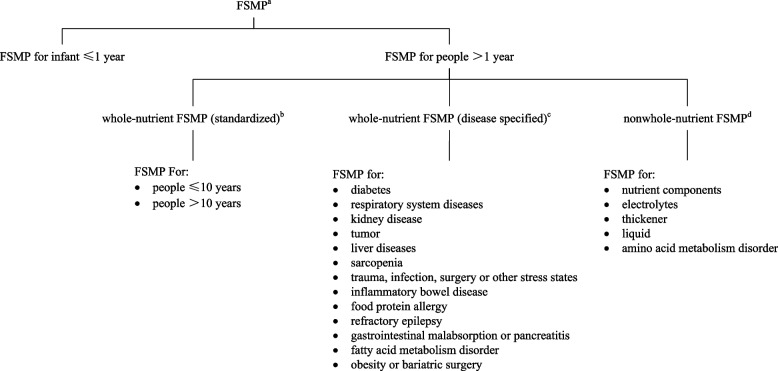
FSMP classification system in China. Superscript lowercase letter a (^a^) indicates the following: FSMP: formula foods that are specially processed and formulated to meet the special needs for nutrients or diets of people with limited eating, digestion, and absorption disorders, metabolic disorders, or specific disease states. Such products should be consumed alone or in combination with other foods under the guidance of a doctor or clinical dietitian. Superscript lowercase letter b (^b^) indicates the following: whole-nutrient FSMP (standardized): can be used as a single source of nutrition to meet the nutritional needs of the target population. Superscript lowercase letter c (^c^) indicates the following: whole-nutrient FSMP (disease specified): can be used as a single nutritional source to meet the nutritional needs of the target population diagnosed with specific diseases or medical conditions. Superscript lowercase letter d (^d^) indicates the following: nonwhole-nutrient FSMP: can meet part of the nutritional needs of the target population but is not suitable for use as a single source of nutrition

Food for Special Medical Purposes (FSMP), also known as medical food in the USA, has been widely used in clinical practice since the 1970s [2]. FSMP has made significant contributions in improving nutritional status, promoting rehabilitation, shortening hospital stays, and saving medical expenses [3–7]. The definition of FSMP and different categories of FSMP are shown in Fig. 1.

However, China entered this field relatively late but passed stricter acts. It has made clinical trials for FSMP compulsory to regulate the domestic FSMP market and achieve the goal of the “Healthy China 2030” initiative [8, 9]. Since 2013, the State Administration for Market Regulation (SAMR), previously known as the China Food and Drug Administration (CFDA), and other government bureaus have gradually issued documentation related to the registration, administration, and supervision of the FSMP. These documentations stipulate that FSMP designed for certain diseases, i.e., whole-nutrient FSMP (disease specified), cannot be registered successfully without clinical trial reports [9–12].

Unbalances have appeared between the supplies and requirements of FSMP. Search results from the Special Food Information Query Platform indicate that 92 FSMP products have been registered; however, only one product has been registered under the category of whole-nutrient FSMP (disease specified) as of November 3, 2022 [13]. Another 39, 23, and 29 FSMP products were registered in the infant FSMP, whole-nutrient FSMP (standardized), and nonwhole-nutrient FSMP segments, respectively.

Additionally, the features of existing clinical trials for FSMP, including long conducting time and diverse outcome selections, may exacerbate the imbalances by slowing down the process of FSMP registration and supplies. Our systematic review (SR) enrolled 46 studies of FSMP designed for T2DM from 14,198 items searched from 6 medical databases. The total intervention duration included in the enrolled studies ranged from 6–90 days, of which the more commonly used durations were 7, 14, 28, 84, and 90 days. Those trials are usually divided into 2, 3, or 4 cycles, with an interval of 2–14 days, but the washout period of 7 days is the most common. After integrating another 9 indices obtained from CFDA files to 161 outcomes extracted from the method sections of the enrolled studies, the outcome pool increased to 170, but only 54 (31.8%) outcomes appeared ≥ 3 times [14, 15]. The frequencies of only 3 outcomes, i.e., fasting blood glucose, postprandial blood glucose, and triglyceride levels, touched half of the enrolled studies (≥ 23 times). Outcomes are so diverse in FSMP fields that research waste occurs, as data deviating from clinical needs cannot be combined or compared [16, 17].

Although diverse outcomes give clinical trials of FSMP freedom to design, they increase the difficulty of registration approval for the government. Therefore, a compact and lightweight solution may better suit governmental needs. Core outcome set (COS) is one alternative solution to compressed outcomes of FSMP to the minimum without losing what is essential or required for each stakeholder group. COS is defined as the minimum sets of outcomes that should be measured and reported [18]. It can help standardize the selection, measurement, and reporting of outcomes, improve the practicality and comparability of research results, and optimize the use of individual research data [17, 19–21]. Therefore, COS will be an optimized option to assess and control risks before and after hitting the market, as applying secondary analysis of data will be feasible.

This study aims to use T2DM as an example to develop COS for clinical trials of FSMP and provide a multistakeholder agreed tool for regulators to use for the pre- and postmarket management of FSMP.

## Methods

The entire study design adopted the approach recommended in COS-STAP and Core Outcome Set-STAndards for Development (COS-STAD), the minimum standard recommendations to improve the methodological approach toward planning a COS study [17, 22, 23]. The results will be reported in the format of the Core Outcome Set–STAndards for Reporting (COS-STAR), a reporting guideline for studies developing COSs [24].

### Scope

1. The health condition(s) and population(s) covered by the COS

This COS is developed for adult T2DM patients who are malnourished or are at nutritional risk; need to receive EN (Enteral Nutrition), without food allergy history such as having symptoms of pruritus or mild cutaneous eruption, etc., after taking milk, eggs, or FSMP; and do not have acute or severe complications, including endocrine diseases such as hyperthyroidism, or other diseases that can significantly influence the estimated energy requirement (EER). They should be capable of taking oral antidiabetic drugs or injecting exogenous insulin. There are no limits to the course of T2DM. Both new and old patients can be included. Women during the gestation or lactation periods will be excluded.

(2) The intervention(s) covered by the COS

The intervention covers FSMP designed for T2DM used in medical nutrition therapy (MNT), which has previously also been known as the elemental diet, monomeric formula, nonelemental diet, and polymeric formula [25]. T2DM patients can fully or partially obtain the required nutrients from these types of FSMP with or without feeding tubes. Blenderized diets are included but not nutrient modules such as peptides, vitamins, minerals, or dietary fibers.

(3) The setting(s) in which the COS is to be applied
  - Research and routine clinical practice.
  - Institutions qualified to conduct clinical trials of FSMP are recommended to use this COS. Detailed information on these qualified institutions can be found on the official website of SAMR [26].

### Stakeholders

A diverse representative sample of stakeholders from a broad geographical area within China will participate anonymously. They should either have COS development experience or have been involved in the research and development, registration, production, supervision, and circulation chain of FSMP. Key stakeholders will consist of (a) COCONUT steering committee members and (b) other stakeholders who will have consecutively participated in all rounds of Delphi surveys. They will be given priority to attend the consensus meeting.

#### Stakeholder groups

Stakeholders will be divided into 4 groups and will be involved in the COS development process at all stages to ensure that the outcomes relevant to all groups are included for the COS to be widely adopted [27]. Some of them are mixed stakeholder representatives. These 4 groups will be as follows:

1. COS developers: They will be recruited from among the corresponding authors of published COS projects in the ChiCOS database and requested to forward the invitation to other authors of those COS projects [28].
2. Journal editors: They will be recruited from the editorial board of the Chinese Journal of Evidence-Based Medicine, etc., in which COS studies have been published.
3. COS users: They will be recruited from (a) researchers of clinical trials enrolled in our SR; (b) nutritional clinical guideline developers; (c) healthcare professionals of clinical nutrition and medicine; (d) regulators from the Special Food Safety Supervision and Administration Department of SAMR; and (e) representative manufacturers in the FSMP industry.
4. Patient representatives: They will be recruited via snowball sampling processes from the Hospital-to-Home Nutrition Management Center (H2H) [29]. Patient inclusion criteria have been described in the scope section.

### COCONUT working group

The working group of Core Outcome sets and Core Outcome measurement sets in NUTriology (COCONUT) refers to the coordinator of this study, and it has been initiating a series of nutriological COS studies since 2019 [30].

#### The steering committee of COCONUT

COCONUT will bring together approximately 60 committee members to consider the COS with the assistance of the Clinical Nutrition Specialty Alliance of West China Hospital, Sichuan University/Western Medical Nutrition Alliance (WMNA) [31], Chinese Gerontological Society of Nutrition and Food Safety Association (CGSN) [32], the Chinese Evidence-Based Medicine Center (Cochrane China Center) [33], and the Chinese Clinical Trials Core Outcome Set Research Center (ChiCOS) [28].

The steering committee will be convened for the following functions:

1. Monitor and review the results of each round of the Delphi survey
2. Attend and help facilitate the consensus meeting
3. Review, finalize, and contribute to the publication and dissemination of the COS, reporting guidance of COS for FSMP, and relevant explanatory documents

#### The expert panel of COCONUT

The panel will include the leader of the steering committee and 3–5 other authoritative committee members. They will review and guide the steering committee at each phase of this project in addition to chairing the consensus meeting.

#### The secretariat of COCONUT

The secretariat comprising COS developers of this study will be convened to perform the following functions:

1. Maintain contact with the stakeholders
2. Conduct SR and Delphi surveys
3. Organize the consensus meeting
4. Acquire, analyze, and interpret the data and draft articles

### Overview

**Fig. 2:**
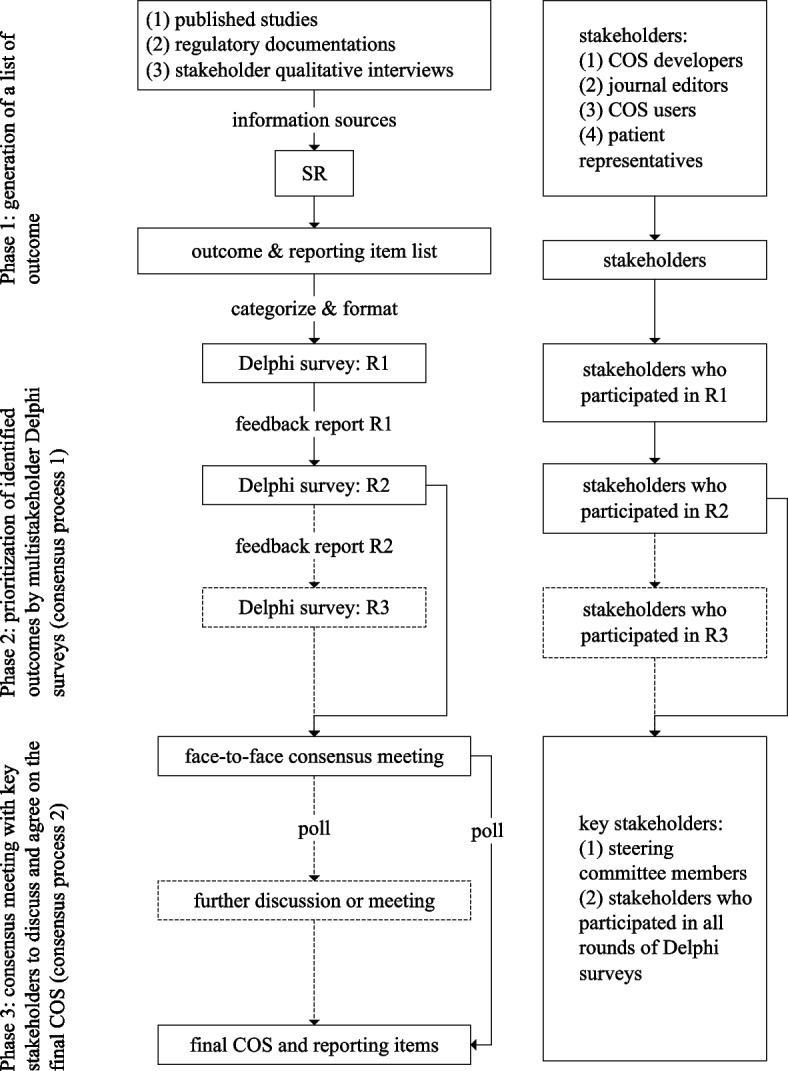
Flowchart of developing the COS of FSMP designed for T2DM

This COS study will have 3 phases, as shown in Fig. 2. Briefly, these would be as follows:

1. Generate a list of relevant outcomes identified from an SR. The information sources of the SR are mainly from (a) published studies, (b) regulatory documentation, and (c) qualitative interviews of stakeholders. Identified outcomes will be categorized via a conceptual framework and formatted into the first round of Delphi survey questionnaire items.
2. Perform at least two consecutive rounds of Delphi surveys among stakeholders invited by the working group of Core Outcome sets and Core Outcome measurement sets in NUTriology (COCONUT) to create the COS for future clinical trials of FSMP. COCONUT will invite patients, clinical dietitians, physicians, COS researchers, journal editors, FSMP manufacturers, and regulatory representatives to score each outcome from the aspects of importance, operability, independence, and cost.
3. Hold a face-to-face or online consensus meeting (if necessary) to refine the COS content. Key stakeholders will be invited to attend the consensus meeting to discuss and reach an agreement regarding the final content of the COS [34].

### Phase 1: Generation of a list of outcomes (information sources)

SR of outcome selection

We will conduct an SR to first identify the preliminary list of outcomes according to the principle of PICOS.

1. PICOS principles
  - Patient/population/problem, P
  As described in the scope section.
  (b) Intervention, I
  As described in the scope section.
  (iii) © Comparator, C
  Comparators can include blanks, standard enteral nutrition (EN) preparations, therapeutic diets, or nutrition education. The use of FSMP designed for T2DM is the only difference between the experimental and control groups.
  (iv) Outcome, O
  Owing to SAMR regulations, outcomes identified from SR will be conceptualized into 4 domains, i.e., safety, nutritional adequacy, special medical effects, and others.
  (e) Study design, S

All original studies related to FSMP designed for T2DM will be enrolled, although RCTs will be preferred. If RCTs do not meet the PICOS criteria, the outcome information will be supplemented by SR, meta-analysis, nonrandomized concurrent controlled studies, and observational studies.

(2) Information sources

The information sources of the SR comprise (a) published studies, (b) regulatory documentation, and (c) qualitative interviews of stakeholders.

Articles related to the subject will be retrieved from 6 medical databases (CNKI, Wanfang, VIP, PubMed, Ovid-Medline, and Cochrane Library). Regulatory documentation related to FSMP will be retrieved from the official SAMR websites and other international regulatory bodies. Online surveys using questionnaires will be performed to collect information from different stakeholders, such as patients, health professionals, FSMP manufacturers, and SAMR officers. Interview surveys will be used as an alternative if online questionnaires are not suitable for every interviewee. Pilot studies to check the readability of questionnaires will be undertaken to ensure that simple language is used and that nonprofessionals can understand the questions.

(3) Search strategies and data selection

Languages will be limited to Chinese and English. No date limitations. The combination of P and I in the PICOS requirements will be selected for the search strategy to avoid omissions. Keywords will contain MeSH terms and synonyms of FSMP, medical foods, EN preparations, and T2DM. Two trained postgraduate students will independently check bibliographies, extract information, evaluate qualities, and record processes. In case of differences between the two students’ decisions, another senior researcher who has relevant experience will evaluate the concerns and determine its inclusion.

(4) Conceptualization of outcomes

The China Food and Drug Administration (CFDA), later replaced in 2018 by SAMR after the First Session of the 13th National People’s Congress, published the Clinical Trial Quality Management Practices (for trial implementation) [12] in 2016, in which the observation outcomes were limited to the domains of (a) safety, (b) nutritional adequacy, and (c) special medical effects. The practice also asks qualified institutions to draw conclusions after thoroughly analyzing and explaining the 3 domains. Therefore, we intend to categorize relevant outcomes into the same 3 domains and another domain, (d) others, in case an outcome identified from the SR cannot be categorized under the first 3 domains.

### Phase 2: Prioritization of identified outcomes using multistakeholder Delphi surveys (consensus process 1)

We will use a consensus process involving a sequential, multiround Delphi survey followed by a face-to-face or online consensus meeting to reach an agreement among multistakeholder groups on the final COS. A diverse representative sample of key stakeholders from the eastern, central, western, and northeastern regions of China will participate anonymously to avoid the effects of dominant individuals. The geographical regions, also called economic regions, were divided by the National Bureau of Statistics of China based on their social and economic development [35].

1. Round 1(R1)

In the first round, stakeholders will be approached by sending out a personalized e-mail with a link to an online questionnaire survey (WJX) [36] with the aid of DelphiManager (Chinese edition), a free software provided by ChiCOS and designed for the Delphi process. Stakeholders will receive background information on the rationale of the development of the COS and the preliminary list of outcomes. Then, they will be asked to provide their basic information, including their name, phone number, fields of expertise, institutions, and stakeholder group categories. Next, they will be suggested to use 9-point Likert scales to score the importance of every outcome in the preliminary list. A score of 1–3 points means not important, 4–6 means important, and 7–9 means critical. Additionally, they will be asked to indicate what outcomes they have newly added. In addition to the Likert scale, an option of “unable to score” is also available in the questionnaire.

(2) Round 2 (R2)

Stakeholders who participate in R1 will then be invited to undertake R2. The R2 questionnaire will contain what is retained from R1 (see the “Analysis” section) and an anonymized feedback report from R1 in the form of summary scores. There will be the option of transforming the distribution of the summary scores into histograms and inserting them into the questionnaire to avoid unnecessary modifications and help establish consensus [37]. If the score is inconsistent with the previous round, the reason for the inconsistency will be needed.

### Phase 3: Consensus meeting with key stakeholders to discuss and agree on the final COS (consensus process 2)

Key stakeholders will be given priority to attend face-to-face or online consensus meetings. One of the COS developers in this study, the leader of the COCONUT steering committee, will chair the meeting, remind the attendees to discuss in simple language, and guide them to poll on every single outcome. The poll results will be simultaneously displayed on the screen in the conference room using a web-based poll program (WJX) [35]. COCONUT secretaries will record the audio of the meeting and transcribe it verbatim. Following the first round of the Delphi survey, outcomes will be categorized as “consensus in,” “consensus out,” or “no consensus” using the definitions in Table 1. Further discussions or meetings will be considered if an agreement is not reached. The same criteria to define consensus and retain/discard outcomes as in Table 1 will be used.

**Table 1:** Definitions of consensus in, consensus out, and no consensus

Category	Consensus in	Consensus out	No consensus
Round 1	Any stakeholder group score as critical (7–9 points) ≥ 70% and not important (1–3 points) < 15%	All stakeholder group score as not important (1–3 points) by ≥ 70% and critical (7–9 points) by < 15%	Neither criterion of consensus in nor out are met
Action 1	Outcome retained for round 2	Outcome discarded before round 2 (to be ratified at consensus meeting)	Outcomes retained for round 2
Round 2	All stakeholder group score as critical (7–9 points) ≥ 70% and not important (1–3 points) < 15%	Any stakeholder group score not important (1–3 points) by ≥ 70% and critical (7–9 points) by < 15%	Neither criterion of consensus in nor out are met
Action 2	Outcome retained for consensus meeting	Outcome discarded before consensus meeting (to be ratified at consensus)	Outcomes retained for consensus meeting
Consensus meeting	All stakeholder group score as critical (7–9 points) ≥ 70% and not important (1–3 points) < 15%	Any stakeholder group score not important (1–3 points) by ≥ 70% and critical (7–9 points) by < 15%	Neither criterion of consensus in nor out are met
Action 3	Outcome retained	Outcome discarded	Outcomes retained for the next consensus meeting

### Sample size

The expert panel was born from the steering committee. The stakeholders comprise the steering committee (professionals, stakeholder groups 1–3) and patients (nonprofessionals, stakeholder group 4). A 3:1 ratio of professionals to patient participants is considered appropriate, as the involvement of multiple professional stakeholder subgroups is warranted in the development of COS [34, 37]. Approximately 13–15 more stakeholders are added to account for the possibility of 10% of stakeholders dropping out during the Delphi processes. Therefore, we aim to use purposive and snowball sampling to include approximately 100 professional participants and 30 patient participants, of whom 15–25 key stakeholders will be invited to attend the consensus meeting. Key stakeholders will comprise (a) steering committee members and (b) stakeholders who will have consecutively participated in all rounds of Delphi surveys.

### Analysis

#### Outcome scoring/feedback

Detailed information is described in phase 2.

#### Missing data

1. Maximizing completion

To keep the dropout rate as low as possible (preferably less than 20%), the language of the questionnaires should be revised to a degree that all stakeholder groups can easily understand. Moreover, the reliability and validity of questionnaires will be tested in a small group of people before use (presurvey, approximately 15 professionals and 5 patients). Text boxes will be inserted at the end of questionnaires, where interviewers can express their thoughts freely, which will help gain more related information.

To increase the response rate, questionnaires will be sent with a brief background introduction containing the aim of developing the minimum core set and an official reference letter at the beginning of the email. WeChat messages will be sent as notices at the same time an email is sent. The feedback report of the previous round will consist of a summary of what has been done, including retain outcomes, discard outcomes, and any other adjustments. We will then personalize the feedback report to avoid biases in which stakeholders can only receive information concerning themselves and their stakeholder group.

(2) Data quality control

Each round of questionnaires will be issued and retrieved by email using the DelphiManager (Chinese edition). Phone numbers and email addresses of the main researchers will be attached to the first page of the questionnaire to answer any questions that stakeholders may have while filling out the questionnaire. If some fields are missed or incorrectly filled, the corresponding stakeholders will be asked to refill them and ensure that each field is complete. If the score is inconsistent with the previous round, reasons should be attached to the changed score. The last edition of the email sent by the stakeholders in every round will be downloaded, printed, and coded, and both paper and electronic forms will be filed.

(3) Data analysis

DelphiManager (Chinese edition) and IBM SPSS 22.0 will be used for data analysis. Questionnaires with omitted values will not be included in the final analysis.

## Discussion

### 9-point or 3-point Likert scale

Many COS studies choose a 9-point Likert scale as their score method, but there have been COS studies involving traditional Chinese medicine indicating that the 3-point Likert scale may be better suited to the Chinese language environment than the 9-point Likert scale [38]. If the 9-point Likert scale does not work well in the presurvey, i.e., scores of 2, 5, and 8 account for more than 80% of all scores, we will consider replacing the 9-point Likert scale with a 3-point scale.

### Further round of Delphi surveys

Another round (*R*_*n*_, *n* ≥ 3) of the Delphi survey will be conducted if significant numbers, i.e., more than 50% of outcomes, remain in the outcome pool after *R*_*n*-1_. The methods in *R*_*n*_ will be identical to those in *R*_*n*-1_. The outcomes remaining after *R*_*n*-1_ and the feedback report from *R*_*n*-1_ will be included in the *R*_*n*_ questionnaire. Stakeholders who have consecutively taken part in *n*-1 rounds of Delphi surveys will rescore again. The outcomes remaining after the final Delphi survey round will be taken forward to the consensus meeting.

### Streamlined scoring group

COS developers, journal editors, COS users, regulators, and manufacturer representatives will be regarded as one scoring group when conducting statistics if we fail to invite more than 30 persons per subgroup. Patient representatives will be regarded as another scoring group.

### Alternative plan of consensus definition

If the consensus definition in Table 1 failed to discard 10% of outcomes in R1. We will consider starting an alternative plan (Table 2) or combining the consensus definition in Table 1 and the alternative plan. A detailed alternative plan description can be found in Additional file 1.

**Table 2:** Alternative consensus definitions

Category	Added/retained	Deleted directly	Deleted indirectly
Round 1	≥ 2 stakeholders agree to add	≥ 2 unqualified scales among M, FR, CV POR ≥ 10% ≥ 2 unqualified scales among M, FR, CV, POR, PIMR, PIDR M ≤ 60% of the full mark or FR ≤ 0.2 in round 1	< 2 unqualified scales among M, FR, CV, POR, PIMR, and PIDR < 2 stakeholders advise to delete
Action 1	Outcome added for R2	Outcome discarded before R2	Outcome discarded before R2 (require expert panel’s approval), otherwise retained for R2
Round 2	Not allowed to newly add outcomes	≥ 2 unqualified scales among M, FR, CV POR ≥ 10% ≥ 2 unqualified scales among M, FR, CV, POR, PIMR, PIDR M ≤ 80% of the full mark or FR ≤ 0.3 in R2	< 2 unqualified scales among M, FR, CV, POR, PIMR, PIDR < 2 stakeholders advise to delete
Action 2	Null	Outcome discarded before consensus meeting	Outcome discarded before consensus meeting (require expert panel’s approval), otherwise retained for consensus meeting
Consensus meeting	All stakeholder group score as critical (7–9 points) ≥ 70% and not important (1–3 points) < 15%	Any stakeholder group score not important (1–3 points) by ≥ 70% and critical (7–9 points) by < 15%	Neither criterion of consensus in nor out are met
Action 3	Outcome retained	Outcome deleted	Outcomes retained for the next consensus meeting

*M* arithmetic mean, *FR* full ratio, *CV* coefficient of variation, *POR* poor operability ratio, *PIMR* poor importance ratio, *PIDR* poor independence ratio

### Key applications

This COS for clinical trials of FSMP will provide a multistakeholder agreed tool for regulators to use for the pre- and postmarket management of FSMP. Premarket, COS will act as a referring guide for manufacturers to produce FSMP in laboratories, allowing them to weed out formulations that do not show effect. The same COS will be used as an assessment criterion in institutions conducting clinical trials of FSMP. Regulators will not approve a product to be registered as FSMP unless its clinical trial reports show acceptable results of COS. Postmarket, real-world data and evidence of COS will help clinical dietitians and doctors choose the best and most suitable FSMP for their patients, providing them with the satisfaction as COS is also what they are concerned about. In summary, COS has the potential to act as a unified metric and communication tool from top to bottom.

If this example of COS proves successful, we could potentially apply the experience to FSMPs designed for other diseases. When COS of FSMPs gains momentum, leading to a series of COSs, it may be time to develop technical specifications, guidelines, or standards. We believe that both the clinical nutrition expertise field and the FSMP industry will benefit from COS processes and become stronger in China.

### Strengths and limitations of this study

1. To develop a COS for the premarket evaluation and postmarket monitoring of FSMP designed for specific diseases, we will combine and use typical methods—SR, Delphi, and consensus—in series, as this guarantees the most possibility of success. According to the latest SR conducted by COMET, 53% of COS research used a mixed method, with Delphi combined with other methods being the most common (54.2%), followed by consensus conferences or SR combined with other methods (14.1%) [39]. Furthermore, our information sources are beyond SR. We will also use stakeholder qualitative interviews to generate a preliminary list of outcomes, providing a broader view of FSMP trial outcomes at the start of phase 2 and helping to avoid missing anything.
2. We will develop COS with multistakeholder groups, including patients, clinical dietitians, physicians, FSMP manufacturers, and policymakers. These five groups are responsible for decisions related to the research and development, registration, production, supervision, and circulation chain of FSMP. Achieving consensus among them is challenging, but any agreement reached will refresh the cognition in the entire FSMP industry and its application in clinical nutrition.
3. This COS may have limited applicability outside of China, as our study is not international. Further work is required to implement COS to FSMP designed for other medical states/diseases.

## Trial status

This study is ongoing at phase 1 (SR has been finished) as planned by the version 1.0 protocol and is expected to be completed by the end of 2024.

## Supplementary Information

**Additional file 1: **Detailed methods description of the alternative consensus definition plan.

## Data Availability

The corresponding author has access to the final trial dataset and discloses contractual agreements that limit such access for investigators.

## Abbreviations

BMI: Body mass index
BW: Body weight
CC: Calf circumference
CGSN: Chinese Gerontological Society of Nutrition and Food Safety Association
ChiCOS: The Chinese clinical trials core outcome set research center
COCONUT: The working group of Core Outcome sets and Core Outcome measurement sets in NUTriology
COS: Core outcome set
COS-STAD: The Core Outcome Set-STAndards for Development
COS-STAP: The Core Outcome Set-STAndardised Protocol Items
CV: Coefficient variation
EER: Estimated energy requirement
EN: Enteral nutrition
FR: Full ratio
FSMP: Food for special medical purposes
H2H: Hospital-to-Home Nutrition Management Center
M: Means
MAC: Middle arm circumference
MNT: Medical nutrition therapy
ONS: Oral nutritional supplements
POR: Poor operability ratio
PIMR: Poor importance ratio
PIDR: Poor independence ratio
SAMR: The State Administration for Market Regulation
SR: Systematic review
T2DM: Type 2 diabetes mellitus
WMNA: The Clinical Nutrition Specialty Alliance of West China Hospital, Sichuan University/Western Medical Nutrition Alliance
