# Supplementary material for: Development of core outcome sets of Food for Special Medical Purposes designed for type 2 diabetes mellitus: a study protocol
Source: Trials. 2023 Mar 24;24:223. doi: 10.1186/s13063-023-07214-2 (PMC10037765; doi:10.1186/s13063-023-07214-2)
Supplement: Supplementary file 1 — Additional file 1: Detailed methods description of the alternative consensus definition plan. [file 13063_2023_7214_MOESM1_ESM.docx]

### Additional files

#### Additional file 1 detailed methods description of the alternative consensus definition plan

##### Boundary value method

During Delphi surveys, stakeholders will be asked to score every outcome in aspects of importance, independence, and operability., i.e., one stake holder will give one outcome 3 scores. Then, we will summarize the scores and calculate the arithmetic mean (M), full score ratio (FR), coefficient of variation (CV) and their boundary values. The boundary value of M or FR is calculated as the mean minus the standard deviation, while the boundary value of CV is the sum of the mean and the standard deviation. If the M of an outcome is lower than the boundary value, it will be recorded as 1 unqualified scale. Accordingly, if the FR is lower than the boundary value or CV is higher than the boundary value, it will also be recorded as 1 unqualified scale.

###### Arithmetic mean (M)

The greater M is, the better the comprehensive evaluation result of the corresponding outcome.

$$\bar{x_{j}}=\frac{1}{m_{j}}\sum_{i=1}^{m} C_{ij}$$

(Formula 1)

where $\bar{x_{j}}$ indicates the average score of the outcome $j$ by the stakeholders; $m_{j}$ indicates the number of stakeholders participating in the scoring of the outcome $j$; and $C_{ij}$ indicates the score of the stakeholder $i$ on the outcome $j$.

###### Full score ratio (FR)

The value of FR is between 0 and 1. The greater the FR is, the higher the proportion of stakeholders who give full marks to the outcome, and the higher the degree of acceptance of the outcome.

$$K_{j}=\frac{m_{j}^{'}}{m_{j}}$$

(Formula 2)

where $K_{j}$ represents the FR of the outcome $j$; $m_{j}^{'}$ indicates the number of stakeholders who give a full score for the outcome $j$; and $m_{j}$ indicates the whole number of stakeholders who participate in the evaluation of the outcome $j$.

###### Coefficient of variation (CV)

It shows the consistent degree of stakeholders' judgment on a certain outcome, also known as the degree of fluctuation. The smaller the CV is, the higher the degree of consistency of stakeholder judgment.

$$V_{j}=\frac{\sigma_{j}}{M_{j}}$$

(Formula 3)

In the formula, $V_{j}$ is the CV of outcome $j$ judged by stakeholders; $\sigma_{j}$ represents the standard deviation of outcome $j$; and $M_{j}$ represents the average score of outcome $j$.

##### Expert scoring method (nonconformance ratios, POR/PIMR/PIDR)

Poor ratios are the proportion that the stakeholders give a certain outcome a "poor" score (1-3 points). The poor ratios of importance, operability, and independence are called the poor operability ratio (POR), poor importance ratio (PIMR), and poor independence ratio (PIDR). If one of POR, PIMR or PIDR of an outcome is more than 10%, it will be recorded as one unqualified scale.

$$P=\frac{m_{i}}{m}\times100\%$$

(Formula 4)

In the formula, $m$ is the whole number of stakeholders, and $m_{i}$ is the number of stakeholders who give a certain outcome a "poor" score.
